# Three Cases of Previous Smokers with Rheumatoid Arthritis Who Did Not Respond to Tumor Necrosis Factor Inhibitors Were Treated Successfully with an Anti-Interleukin-6 Receptor Antibody

**DOI:** 10.1155/2015/806725

**Published:** 2015-01-14

**Authors:** Yasuo Iwata

**Affiliations:** Iwata Orthopedics and Rheumatology Clinic, No. 13-5, 1A Nakasuji 8-Chome, Takarazuka, Hyogo 665-9874, Japan

## Abstract

We report three cases of previous smokers who did not respond to TNF inhibitors but who responded successfully to an anti-interleukin-6 receptor antibody (tocilizumab (TCZ)). Case 1 is a 63-year-old woman whose smoking index was 200 and had been complaining of polyarthralgia since 1996. She started treatment with etanercept due to high disease activity, but her DAS28-CRP was 4.2. She was therefore switched to TCZ, which dramatically improved her symptoms; her DAS28-CRP had decreased to 2.1. Case 2 is a 64-year-old man whose smoking index was 1600 and had been complaining of polyarthralgia since 2006. Because his DAS28-CRP score increased over time to 5.9, etanercept and adalimumab were added sequentially, but he showed no response over the course of two years. The patient was therefore switched to TCZ, which dramatically improved his symptoms: his DAS28-CRP decreased to 2.7. Case 3 is a 48-year-old woman whose smoking index was 560 and had been complaining of pain in both knee joints since 2001. She was treated with adalimumab due to high disease activity but showed no response over the course of 1.5 years. The patient was therefore switched to TCZ, and her DAS28-CRP decreased to 1.8. An IL-6 blockade might be suitable for treating these 3 cases of previous smokers.

## 1. Introduction

Tumor necrosis factor (TNF) inhibitors represent an important advance in therapy for rheumatoid arthritis (RA). RA patients who smoke, however, are reported to be less likely to respond to treatment with TNF inhibitors [1–4]. This report presents three cases of smokers who did not respond to TNF inhibitors but who responded successfully to an anti-interleukin-6 receptor antibody (tocilizumab [TCZ]).

## 2. A Case Report

**Figure 1:**
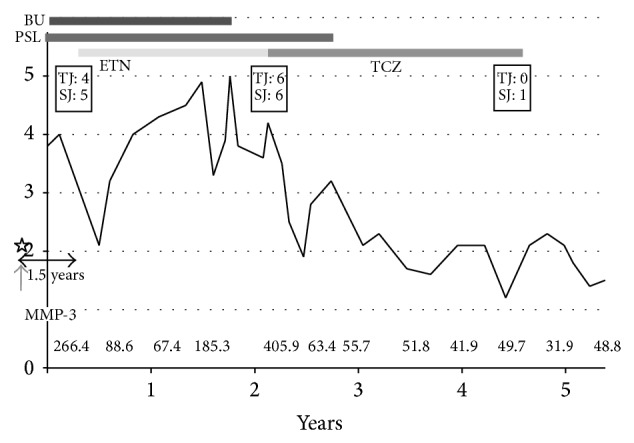
Summary of the clinical course of case 1. DAS28-CRP Disease Activity Score assessing 28 joints with C-reactive protein. SASP: salazosulfapyridine, PSL: prednisolone, ETN: etanercept, TCZ: tocilizumab, and MMP-3: matrix metalloproteinase-3. TJ means tender joint counts and SJ means swollen joints counts for the assessment of DAS 28-CRP. The asterisk shows the cessation of smoking. Etanercept was initiated 1.5 years after the cessation of smoking.

Case 1 is a 63-year-old woman whose smoking index was 200 (10 cigarettes/day × 20 years) (Table 1) and had been complaining of polyarthralgia since 1996. She could not take methotrexate due to the adverse effects of liver dysfunction and hair loss. During treatment for RA, she was able to quit smoking as per our instructions. Two years after her first visit, the lateral tibial condyle of her right knee joint collapsed. As a result, she underwent total knee arthroplasty. She started treatment with the TNF inhibitor etanercept due to high disease activity (Disease Activity Score assessing 28 joints with C-reactive protein [DAS28-CRP] was 4) 1.5 years after cessation of smoking but showed no response. Two years after starting this medication, her DAS28-CRP was 4.2 and her MMP-3 was 405 ng/mL. The patient was therefore switched to TCZ (8 mg/kg monthly), which dramatically improved her symptoms. Six months after switching to TCZ, her DAS28-CRP had decreased to less than 2.3 and her MMP-3 had decreased from 405 to less than 59.7 ng/mL (Figure 1). She has satisfied the Boolean-based definition for over 10 months after the cessation of the TCZ therapy. Recent radiograms of the involved joints show nonprogression.

**Figure 2:**
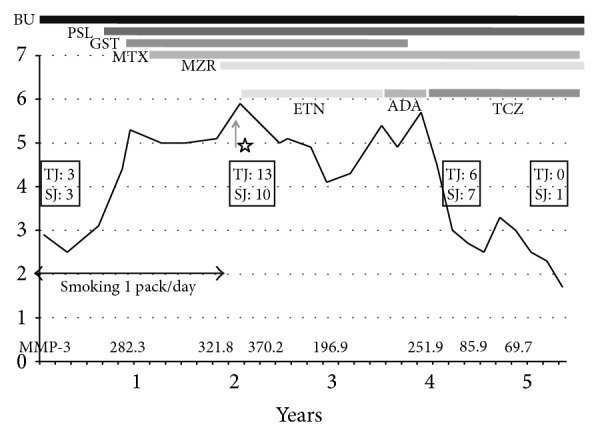
Summary of the clinical course of case 2. DAS28-CRP Disease Activity Score assessing 28 joints with C-reactive protein. BU: bucillamine, PSL: prednisolone, GST: gold sodium thiomalate, MTX: methotrexate, MZR: mizoribine, ETN: etanercept, ADA: adalimumab, TCZ: tocilizumab, and MMP-3: matrix metalloproteinase-3. The asterisk shows the cessation of smoking. TJ means tender joint counts and SJ means swollen joints counts for the assessment of DAS 28-CRP.

Case 2 is a 64-year-old man whose smoking index was 1600 (40 cigarettes/day × 40 years) (Table 1) and had been complaining of polyarthralgia since 2006. He did not respond to a combination of methotrexate (8 mg/week), prednisolone (10 mg/day), bucillamine (200 mg/day), and intramuscular injections of gold sodium thiomalate (10 mg/week). During treatment for RA, because his DAS28-CRP score increased over time to 5.9 and because he developed active synovitis of the cervical vertebra, etanercept (50 mg/week) was added to his medications one month after he quit smoking as per our instructions, but the patient showed no response over the course of one year. The etanercept was then replaced with adalimumab (40 mg/2 weeks), but the patient still had no response. Four months after adalimumab was started, his DAS28-CRP was 5.7 and his MMP-3 was 251.9 ng/mL. The patient was therefore switched to TCZ (8 mg/kg monthly), which dramatically improved his symptoms. After switching to TCZ, his DAS28-CRP decreased to less than 2.3 and his MMP-3 decreased to 85.9 ng/mL, but his global assessment ranged from 4 to 5 cm (Figure 2). Recent radiograms of the involved joints show no erosive progression.

**Figure 3:**
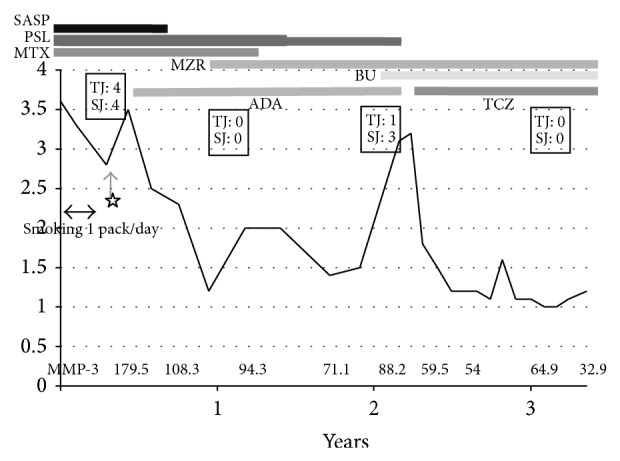
Summary of the clinical course of case 3. DAS28-CRP Disease Activity Score assessing 28 joints with C-reactive protein. SASP: salazosulfapyridine, PSL: prednisolone, MTX: methotrexate, MZR: mizoribine, BU: bucillamine, ADA: adalimumab, TCZ: tocilizumab, and MMP-3: matrix metalloproteinase-3. The asterisk shows the cessation of smoking. TJ means tender joint counts and SJ means swollen joints counts for the assessment of DAS 28-CRP.

Case 3 is a 48-year-old woman whose smoking index was 560 (20 cigarettes/day × 28 years) (Table 1) and had been complaining of pain in both knee joints since 2001. She was initially treated with a combination of prednisolone (10 mg/day) and methotrexate (8 mg/week) but did not respond to these medications despite the fact that she was simultaneously undergoing smoking cessation treatment. She was then switched to treatment with adalimumab (40 mg/2 weeks) due to high disease activity 2 months after she quit smoking but showed no response to the new medication over the course of 1.5 years. At the end of this period, her DAS28-CRP was 3.2 and her MMP-3 was 88.2 ng/mL. The patient was therefore switched to TCZ (8 mg/kg monthly), which dramatically improved her symptoms. After a single drip infusion of TCZ, her DAS28-CRP decreased to 1.8 and her MMP-3 decreased to 59.5 ng/mL (Figure 3). She has since satisfied the Boolean-based definition for 5 months. The latest radiographic examination of the involved joints showed no progression of bone erosion.

## 3. Discussion

Papadopoulos defined a previous smoker as a person who had stopped smoking for at least one year [5]. Cases 2 and 3 were treated with biologics in less than 3 months after smoking cessation; these two cases could therefore be considered current smokers. For case 1 who had smoked for 20 years, the influence of smoking was assumed to exist although etanercept was initiated 1.5 years after quitting smoking. It is said that even if quitting smoking can be achieved, it takes at least 10 years until the influence of smoking disappears from the body [6]. Karlson et al. reported that duration of smoking was associated with a significantly increased risk of developing RA but that smoking intensity (number of cigarettes/day) was unrelated to risk of RA [7].

In case 3, DAS 28-CRP might get better with the use of mizoribine, but the clinical course showed a relapse of arthritis with the use of mizoribine and, after a single drip infusion of TCZ, her DAS28-CRP decreased to 1.8. It is reasonable to assume that TCZ was effective for her RA.

Anti-TNF-alpha agents were reported to have been less effective for the treatment of RA in current and previous smokers [1–4]. The three cases presented herein were also refractory to anti-TNF agents, although the patients had stopped smoking before the administration of anti-TNF agents. The SAMURAI study demonstrated efficacy in patients with RA treated with TCZ monotherapy [8]. Patients with multidrug refractory adult onset Still's disease (AOSD) were successfully treated with TCZ [9–13].

The relationship between cigarette smoking and the improved response to TCZ probably likely has several explanations. In terms of the relation between smoking and periodontitis, smoking is a major risk factor for periodontitis [14–16]. Sites from refractory patient with periodontitis produced significantly more IL-6 [17]. Fibroblasts from periodontal lesions in vitro produce greater amounts of IL-6 and IL-8 constitutively than healthy controls [18]. In periodontitis stroma, increased citrullinated protein presence (80%) was observed compared with control stroma (33%). Western blotting with monoclonal (F95) antibody to citrullinated proteins revealed the presence of similar citrullinated proteins in both periodontitis and RA-affected synovial tissue [19]. These three cases did not complain of oral problems. There are some reports on the relationship between IL-6 in the lungs and smoking. Higher IL-6 and exhaled carbon monoxide (CO) concentrations were found in the exhaled breath condensate of smokers than in that of nonsmokers. In addition, there was a correlation between IL-6 concentrations, number of cigarettes smoked per day, exhaled CO, leukotriene, and lung function [20]. In bronchoalveolar lavage (BAL), statistically greater concentrations of neutrophils, macrophages, IL-1 beta, IL-6, IL-8, and monocyte chemotactic protein-1 (MCP-1) were observed among smokers compared with nonsmokers [21]. Smoking increases peptidylarginine deiminase 2 enzyme expression in the human lungs and increases citrullination in BAL cells [22]. Snelgrove reported that cigarette smoke selectively inhibited leukotriene A(4) hydrolase aminopeptidase activity, which led to the accumulation of the neutrophil chemoattractant proline-glycine-proline and neutrophils, and made inflammation become chronic such as chronic obstructive pulmonary disease (COPD) and cystic fibrosis [23]. Cigarette smoke induces proinflammatory cytokines and chemokines, including IL-1 beta, and IL-6 from synovial fibroblast-like cells (SFCs) [24]. And blood leukocytes, platelets, C-reactive protein (CRP), and fibrinogen were reported to be significantly high in smokers [25]. Mean platelet volume increased significantly with acute exposure to smoking [26]. IL-6 stimulates thrombopoiesis through thrombopoietin [27], and IL-6 activates platelets [28]. Boilard et al. surveyed the capacity of collagen-stimulated human platelet microparticles (MPs) to elicit a range of cytokines and chemokines from fibroblast-like synoviocytes (FLSs) and observed prominent production of the broadly inflammatory cytokine IL-6 and the neutrophil chemoattractant IL-8 without production of TNF alpha [29]. Therefore, the positive feedback of IL-6 through fibroblast-like synoviocytes and platelets is formed in the human body. In this case report, cases 1 and 3 showed elevation of platelet count.

Smoking is an established risk factor of RA and may cause prominent production of cytokines especially IL-6 (as described above). An IL-6 blockade might be suitable for treating these 3 cases. But this is a case report; however, further study of a large series of cases is required to determine the efficacy of an IL-6 blockade for patients with RA who smoke.

## Figures and Tables

**Table 1:** Characteristics of patients.

	Case 1	Case 2	Case 3
Sex	Female	Male	Female

Age (years)	63	64	48

Disease duration (years)	12	6	8

Smoking index	200 (10 cigarettes/day × 20 years)	1600 (40 cigarettes/day × 40 years)	560 (20 cigarettes/day × 28 years)

2010 ACR/EULAR classification criteria	Satisfied	Satisfied	Satisfied

Laboratory results	RF 73.8 U/mL ACPA 4.4 U/mL CRP 2.9 mg/dL WBC 11500/*μ*L MMP-3 698.7 ng/mL Platelet 37.1 × 10^4^/*μ*L	RF 60.0 U/mL ACPA 150.0 U/mL CRP 1.5 mg/dL WBC 8600/*μ*L MMP-3 148.1 ng/mL Platelet 35.0 × 10^4^/*μ*L	RF 26.0 U/mL ACPA 128.6 U/mL CRP 0.07 mg/dL WBC 12400/*μ*L MMP-3 179.5 ng/mL Platelet 42.1 × 10^4^/*μ*L

Steinbrocker's roentgenographic classification	Stage IV	Stage III	Stage III

Functional status according to Steinbrocker's revised criteria	Class II	Class II	Class II

Previous treatment: type and dosage (duration in months)	Etanercept 50 mg/week (26) Prednisolone 3 mg/day (62) Bucillamine 200 mg/day (52)	Etanercept 25–50 mg/week (13) Adalimumab 40 mg/2 weeks (4) Methotrexate 8 mg/week (72) Prednisolone 5 mg/day (36) Bucillamine 200 mg/day (48) Gold sodium thiomalate 10 mg/week (24) Mizoribine 150 mg/day (18)	Adalimumab 40 mg/2 weeks (4) Methotrexate 6 mg/week (72) Prednisolone 9 mg/day (36) Salazosulfapyridine 1000 mg/day (6) Mizoribine 200 mg/day (18)

Time (months) to remission of arthritis (DAS28-CRP <2.3)	11	16	1

RF: rheumatoid factor; ACPA: anti-cyclic citrullinated peptide antibody; CRP: C-reactive protein; WBC: white blood cell count; MMP-3: matrix metalloproteinase-3.
